# Adulticidal and repellent activities of traditionally used Ethiopian plants against *Anopheles arabiensis* and *Aedes aegypti*

**DOI:** 10.1186/s41182-026-01029-y

**Published:** 2026-07-15

**Authors:** Lensa Tesfaye, Esayas Aklilu, Ketema Tolossa, Abebe Animut

**Affiliations:** Aklilu Lemma Institute of Health Science Research, Addis Ababa University, Addis Ababa, Ethiopia

**Keywords:** *An. arabiensis*, *Ae. aegypti*, Adulticidal activity, Repellency, Essential oils, Plant extract, Ethiopia

## Abstract

**Background:**

The widespread development of synthetic insecticide-resistant vectors threatens the effectiveness of malaria and arboviral disease control programs. Plant-derived bioactive compounds represent potential alternatives or complements for mosquito vector management.

**Objective:**

Adulticidal and repellent activities of crude methanol extracts and essential oils from selected Ethiopian medicinal plants against *Anopheles arabiensis* and *Aedes aegypti* were evaluated.

**Methods:**

Crude methanolic extracts and essential oils from selected Ethiopian medicinal plants were evaluated for their adulticidal and repellent activities against *Anopheles arabiensis* (*An. Arabiensis*) and *Aedes aegypti* (*Ae. Aegypti*). The insecticidal activity of the crude extracts was tested at concentrations ranging from 13.16 to 52.63 mg/L after 24 h of exposure. Mortality concentration was compared using one-way ANOVA, and the LC₅₀/LC₉₀ ratio was estimated using probit analysis. The repellent efficacy of essential oils (2.5–10%) was assessed via an arm-in-cage method, with percent repellency, complete protection time, and protection duration analyzed via Kaplan–Meier survival analysis.

**Result:**

Extracts of *Agave sisalana* and *Momordica foetida* induced 80% and 79% mortality in *An. arabiensis* at 52.63 mg/L (LC₅₀ = 21.8 and 15.3 mg/L, respectively). *Securidaca longepedunculata* and *Millettia ferruginea* exhibit moderate adulticidal activity against *Ae. aegypti*, with mortality rates of up to 82% and 71%, respectively, at the highest tested concentrations. During brief exposures, essential oils from *Ficus thonningii*, *Securidaca longepedunculata*, and *Momordica foetida* suppressed mosquito bites by up to 79%, 86%, and 80%, respectively, although complete protection lasted only 7 min. Dose–response curves for the most active extracts were statistically significant (*p* < 0.05).

**Conclusion:**

Crude methanol extracts of *Agave sisalana*, *Momordica foetida*, *Securidaca longepedunculata*, and *Millettia ferruginea* exhibit potent adulticidal effects against *An. arabiensis* and *Ae. aegypti*. The essential oils from *Ficus thonningii*, *Momordica foetida*, and *Securidaca longepedunculata* provided short-term repellency. These plant-derived products hold promise as eco-friendly alternatives for mosquito control in Ethiopia, necessitating further studies on their bioactive compounds, formulations, safety, and field efficacy.

**Supplementary Information:**

The online version contains supplementary material available at 10.1186/s41182-026-01029-y.

## Background

Mosquito-borne diseases (MSDs) pose a major public health challenge in sub-Saharan Africa, including Ethiopia [1]. From January to October 2024, Ethiopia reported more than 7.3 million cases and 1157 deaths due to malaria. The disease is caused mainly by *Plasmodium falciparum* (*P. falciparum*) and transmitted by *An. Arabiensis*. These malaria cases and deaths were the highest in the last seven years' records [2]. Approximately 75% of the landmass is malaria endemic, affecting 69% of the population [1]. Dengue outbreaks have been reported in Ethiopia since 2013, and peak transmission rates were observed in the urban settings of Afar and Dire Dawa from 2023–2024; these transmission rates were transmitted by *Ae. aegypti* [3, 4].

Despite the widespread use of insecticide-treated nets (ITNs) and, in some cases, indoor residual insecticide spraying (IRS), mosquito populations continue to adapt and persist [5]. In Ethiopia, *Anopheles* and *Aedes* species exhibit high resistance to pyrethroids, dichlorodiphenyltrichloroethane (DDT), and organophosphates, with resistance exceeding 80% in major malaria-endemic areas, such as the Oromia and Gambella regions. Agricultural pesticide pressure and suboptimal insecticide dosing might exacerbate insecticide resistance in vectors [6].

Rising insecticide resistance, environmental harms, and low community adherence create an urgent need for sustainable, community-centered mosquito-control options [7]. Indoor residual insecticide spraying and ITNs threaten non-target species and aquatic ecosystems, and improper disposal worsens water pollution in Ethiopia [8]. Effectiveness is further reduced by user discomfort, perceived failure due to resistance, and cultural preferences for traditional methods, with ITN use remaining below 50% in some areas [9, 10]. Plant-based repellents and insecticides from local flora (for example, neem and Ocimum) offer environmentally friendly protection, align with community knowledge, and may reduce selection pressure for resistance. When combined with environmental management (such as larval source reduction), these approaches can support national goals for malaria and arboviral control [11, 12].

Rural Ethiopian communities have long used indigenous plants to repel or kill mosquitoes, drawing on cultural heritage, empirical observation, and intergenerational knowledge transmission [13]. However, this ethnobotanical knowledge is often under-documented, poorly validated, and threatened by lifestyle changes. Interest in plant-based mosquito control is growing [14], and Ethiopia’s rich biodiversity and traditional medicine provide promising sources of mosquitocidal and repellent compounds [15, 16]. The selected species are widely used in traditional medicine and contain bioactive secondary metabolites—alkaloids, flavonoids, terpenoids, and essential oils that have shown insecticidal and repellent activity against mosquito vectors in numerous studies [14, 17].

Arjo Gudatu District in western Ethiopia has diverse ecology and rich ethnobotanical knowledge, but traditional mosquito-control plants are poorly documented and rarely experimentally tested. Few Ethiopian species have been validated against adult mosquitoes or compared with standard insecticides and repellents [18]. This study addresses that gap by evaluating the adulticidal and repellent activities of plant species used in Arjo Gudatu, testing them against *An. arabiensis* and *Ae. aegypti* to support integration of local knowledge into vector-control strategies.

## Materials and methods

### Plant collection and processing

Twelve plant species from 12 families, selected based on ethnobotanical knowledge from the Arjo Gudatu district for traditional mosquito control [19], were collected from three kebeles (Lalisa Dimtu, Karsa Dako, and Mada Jalala) between December 2024 and February 2025. The district lies 1100–2300 m above sea level, with a mean annual rainfall of 1400 mm and temperatures of 18–32 °C. Specific parts (leaves, bark, or roots) were harvested (Table 1). A professional botanist authenticated all the samples; voucher samples (L01–L12) were deposited at the National Herbarium, Addis Ababa University, Ethiopia.

**Table 1:** Plant species screened for adulticidal activity in the Arjo Gudatu district, Oromia, Ethiopia

Family name	Scientific name	Vernacular name (Afan Oromo)	Voucher no.	Growth form	Part of the used
Euphorbiaceae	*Croton macrostachyus Del*	Bekenisa	L11	Tree	Leaf
Moraceae	*Ficus thinning Blume*	Bifti	L03	Tree	Bark
Combretaceae	*Terminalia schimperiana Hochst*	Debeka	L05	Tree	Bark
Acanthaceae	*Justicia Schimperiana (hochst.ex Nees) T Ander*	Dhumuga	L10	Herbs	Leaf
Proteceae	*Faurea speciosa Welw*	Garree	L06	Tree	Bark
Agavaceae	*Agave sisalana Perrine ex Engel*	Kacaa	L08	Herbs	Leaf
Asteraceae	*Echinops sphaerocephalus L*	Kebericho	L02	Herbs	Bark
Polygalaceae	*Securidaea longepedunculata*	Tebenaye	L04	Tree	Root
Anacardiadceae	*Lannea schimperi (A Rich) Engl*	Warake	L01	Tree	Bark
Cucurbitaceae	*Momodica foetida schumach*	Imbayoo	L09	Herbs	Leaf
Fabaceae	*Millettia ferruginea (Hochst.ex nees) tander*	Sotolo	L07	Tree	Leaf
Compositeae	*Guizotia scabra*	Hadaa	L12	Herbs	Leaf

Source: Adapted from [19]

### Preparation of the crude plant extracts

The plant parts were washed with distilled water, air-dried in the dark at room temperature for 10 days, and ground into a fine powder using an electric grinder. The powders were stored in airtight containers at room temperature until extraction. The sample powder (50 g each) was macerated in 250 ml of 80% (v/v) methanol for 72 h at room temperature with intermittent shaking on an orbital shaker, filtered through Whatman No. 1 filter paper, and centrifuged at 1500 rpm for 5 min for further clarification of the supernatant. The resulting mixture was concentrated under reduced pressure at 60 °C via a rotary evaporator, after which the residual solvent was further dried in an oven at 40 °C to obtain a dry crude extract. The dried crude extracts were weighed, their percentage yield was calculated, and the samples were stored at 4 °C until they were used for bioassays.

### Extraction of essential oils

Four fresh plant materials (1000 g each) were subjected to hydrodistillation in a Clevenger-type apparatus for 3 h. These species were selected because they were the most frequently cited by local community members for mosquito repellent during the ethnobotanical survey [19], and some plants presented relatively measurable adulticidal activity in preliminary assays. The extracted essential oils were dried over anhydrous sodium sulfate to remove residual moisture and stored in amber glass vials at 4 °C until use in the bioassays [20].

### Mosquito rearing

Laboratory colonies of *An. arabiensis* and *Ae. aegypti* mosquitoes maintained at the Center for Pathobiology Research, Aklilu Lemma Institute of Health Research, Addis Ababa University, Ethiopia, were used for this study. The plants were maintained at 27 ± 2 °C, 70–80% relative humidity, and a 12:12 h light‒dark photoperiod to ensure the reliability and reproducibility of the data. Larvae were reared in dechlorinated tap water and fed yeast daily, and pupae were transferred to emergence cages (60 × 60 × 60 cm). Adults were provided with a 10% sucrose solution. Non-blood-fed adult female mosquitoes aged 3–5 days were used for the adulticidal assays, and those aged 3–7 days were used for the repellency assays.

### Adulticidal bioassay

The efficacy of the extracts was evaluated according to WHO guidelines for insecticide susceptibility testing [21]. Filter papers (12 × 15 cm) were impregnated with 3 ml of test solution at concentrations of 13.16, 26.32, and 52.63 mg/L. The corresponding approximate surface doses were 0.00022, 0.00044, and 0.00088 mg/cm^2^, respectively, and air-dried for 24 h. Papers treated with 5% DMSO served as negative controls, and those treated with 0.03% deltamethrin were used as positive controls. Twenty-five 3- to 5-day, non-blood-fed female *An. arabiensis* and *Ae. aegypti* were introduced into exposure tubes and exposed to treated papers for 1 h. After exposure, the mosquitoes were transferred to holding tubes and provided with 10% sucrose solution. Mortality was recorded after 24 h. Each treatment was performed in triplicate.

Adult mortality (%) was calculated as follows:

$${\text{Mortality }}\left( \% \right) = ({\text{numberof dead mosquitoes}}/{\mathrm{totalexposed}}) \times {1}00.$$

### Repellent bioassay (arm-in-cage method)

Repellent efficacy was evaluated via a modified WHO arm-in-cage procedure [20]. The essential oils were diluted to 2.5%, 5%, and 10% (v/v) ethanol, with 10% *N*,*N*-diethyl-meta-toluamide (DEET) and absolute ethanol used as positive and negative controls, respectively. Five healthy adult volunteers provided written informed consent after Addis Ababa University Institutional Ethics Review Committee approval. The forearm (25 cm^2^) was cleaned with unscented soap and water. One milliliter of the test formulation was applied evenly and air-dried for 15 min (no ethanol odor was detectable). The treated arm was then inserted into a cage containing twenty-five 3- to 5-day-old, non-blood-fed female *An. arabiensis* and *Ae. aegypti* for 3 min. Mosquito landings were recorded, and percent repellency was calculated as PR% = [(*C* – *T*)/*C*] × 100, where *C* and *T* are the mean landings on the control and test arms, respectively.

Each plant species combination was tested three times per volunteer with five volunteers (*n* = 15 replicates per formulation; 3 × 4 × 2 = 24 formulations; 360 total arm-in-cage tests). Clean cages were used to prevent cross-contamination. All tests were conducted at 27 ± 2 °C and 60–80% relative humidity.

### Determination of the complete protection time

The treated arm was reintroduced into the cage at 30-min intervals until the first confirmed bite occurred. The complete protection time (CPT) was defined as the interval between repellent application and the first bite.

### Preliminary phytochemical screening and thin-layer chromatography analysis

Qualitative phytochemical screening was conducted to detect alkaloids, flavonoids, saponins, tannins, terpenoids, and phenolic compounds via standard procedures [22].

Thin-layer chromatography (TLC) was performed using silica gel plates. The samples were developed in the following two solvent systems [23]:

- *n*-butanol:glacial acetic acid:water (4:1:1, v/v)
- *n*-butanol:chloroform (7:3, v/v).

Spots were visualized under UV light (254 and 366 nm) and sprayed with vanillin-sulfuric acid reagent; no reference standards were used. The retention factor (*R*_f_) values were calculated as:

$$R_{{\mathrm{f}}} = {\text{ distance traveled by the compound}}/{\text{distance traveled by the solvent front}}{.}$$

### Data analysis

The data were analyzed using SPSS version 23. Mortality differences among concentrations were assessed via one-way analysis of variance (ANOVA) followed by Tukey’s post hoc test after confirming normality (Shapiro–Wilk test and residual plots) and homogeneity of variances (Levene’s test). LC₅₀ and LC₉₀ values were estimated using probit regression with 95% confidence intervals. Repellency and CPT were analyzed via Kaplan–Meier survival analysis and compared via log-rank tests, with statistical significance set at *P* < 0.05.

### Ethical approval

Ethical approval was obtained from the Addis Ababa University Institutional Ethics Review Committee (Ref. No. ALIPB IRERC/108/2015/23), and written informed consent was obtained from all volunteers before participation. A preliminary skin patch test was conducted to assess potential allergic reactions to the tested plant essential oils, and all volunteers tested negative before enrollment. During the repellency experiments, the volunteers were continuously monitored for any signs of skin irritation, discomfort, or other adverse reactions throughout the 3–7 h exposure period, and no adverse effects were observed. To minimize potential carryover effects and skin sensitization, a minimum washout period of 7 days was maintained between successive exposure sessions.

## Results

### Adulticidal activity of 80% methanol plant extracts against *Anopheles arabiensis*

As shown in Table 2, all the tested plant extracts demonstrated varying levels of adulticidal activity against *An. arabiensis*, with mortality increasing in a concentration-dependent manner, indicating a clear dose‒response relationship. At the highest concentration (52.63 mg/L), *Agave sisalana (A. sisalana)* presented the highest measurable mortality (80 ± 1.4%), followed by *Momordica foetida (M. foetida)* (79 ± 0.7%), *Millettia ferruginea* (*M. ferruginea*) (78 ± 1.1%), *Securidaca longepedunculata (S. longepedunculata)* (62 ± 1.1%), *Ficus thonningii*), *F. thonningii* (59 ± 1.4%), and *Faurea speciosa* (*F. speciosa)* (24 ± 0.7%). In contrast, *Justicia schimperiana* (*J. schimperiana*), *Croton macrostachyus* (*C. macrostachyus*), *Echinops sphaerocephalus* (*E. sphaerocephalus*)*, Lannea schimperi* (*L. schimperi*), *Terminalia schimperiana* (*T. schimperiana*), and *Guizotia scabra (G. scabra)* presented relatively low mortality rates. One-way ANOVA confirmed significant concentration-dependent effects for all the species (*P* < 0.001), including *J. schimperiana* (*F* (2,6) = 16.6, *P* = 0.004). No mortality was observed in the negative control group (5% DMSO), confirming the absence of solvent-induced effects. The positive control (0.03% deltamethrin) resulted in near-complete mortality, validating the susceptibility of the mosquito population and the reliability of the experimental setup.

**Table 2:** Adulticidal activity of methanol plant extracts against *Anopheles arabiensis*

Plant species	Conc. (mg/L)	Mortality % (mean ± SD)	ANOVA *F* (*df*), *p*-value
*A. sisalana*	13.16	25 ± 0.8ᵃ	*F* (2,6) = 619.7, *p* < 0.001
	26.32	66 ± 1.1ᵇ	
	52.63	80 ± 1.4ᶜ	
*C. macrostachyus*	13.16	21 ± 0.8ᵃ	*F* (2,6) = 205.7, *p* < 0.001
	26.32	40 ± 1.5ᵇ	
	52.63	54 ± 1.1ᶜ	
*E.* *sphaerocephalus*	13.16	14 ± 1.1ᵃ	*F* (2,6) = 118.5, *p* < 0.001
	26.32	15 ± 0.8ᵃ	
	52.63	38 ± 1.1ᵇ	
*F. speciose*	13.16	12 ± 0.7ᵃ	*F* (2,6) = 39, *p* < 0.001
	26.32	15 ± 1.4ᵃ	
	52.63	24 ± 0.7ᵇ	
*F. thonningii*	13.16	16 ± 0.7ᵃ	*F* (2,6) = 89, *p* < 0.001
	26.32	38 ± 1.1ᵇ	
	52.63	59 ± 1.4ᶜ	
*G. scabra*	13.16	6 ± 1.1ᵃ	*F* (2,6) = 34.7, *p* < 0.001
	26.32	12 ± 0.7ᵇ	
	52.63	18 ± 1.1ᶜ	
*J. schimperiana*	13.16	32 ± 0.7^a^	*F* (2,6) = 16.6, *p* < 0.004
	26.32	39 ± 1.4^b^	
	52.63	43 ± 0.8^b^	
*L. schimperi*	13.16	7 ± 0.4^a^	*F* (2,6) = 276.6, *p* < 0.001
	26.32	20 ± 0.7^b^	
	52.63	37 ± 1.9^c^	
*M. ferruginea*	13.16	44 ± 0.7^a^	*F* (2,6) = 537, *p* < 0.001
	26.32	51 ± 0.4^b^	
	52.63	78 ± 1.1^c^	
*M. foetida*	13.16	48 ± 0.7^a^	*F* (2,6) = 168, *p* < 0.001
	26.32	60 ± 0.7^b^	
	52.63	79 ± 0.7^c^	
*S. longepedunculata*	13.16	16 ± 1.5^a^	*F* (2,6) = 304.9, *p* < 0.001
	26.32	25 ± 0.7^b^	
	52.63	60 ± 1.1^c^	
*T. schimperiana*	13.16	44 ± 1.5^a^	*F* (2,6) = 115, *p* < 0.001
	26.32	55 ± 0.8^b^	
	52.63	62 ± 1.1^c^	
Negative control	5% DMSO	0	N/A
Positive control	0.03% deltamethrin	97–98	

Values are expressed as the means ± SDs of three replicates. One-way ANOVA followed by Tukey’s HSD test was used for comparisons. Different lowercase superscripts (a–c) within columns indicate significant differences in concentration on mortality (*p* < 0.05); means sharing the same letter are not significantly different. mg/L = milligrams per liter; SD = standard deviation; *df* = degrees of freedom. N/A = not applicable

### Probit analysis and lethal concentrations against *Anopheles arabiensis*

Probit analysis revealed considerable variation in adulticidal toxicity among the tested plant extracts against *An. arabiensis*. The lowest LC₅₀ was recorded for *M. foetida* (15.3 mg/L; 95% CI: 0.94–24.39), followed by *M. ferruginea* (19.1 mg/L; 95% CI: 4.13–30.67) and *A. sisalana* (21.8 mg/L; 95% CI: 14.8–29.1), indicating measurable potency. Lowest toxicity was evident for *F. thonningii* (LC₅₀ = 39.5 mg/L; 95% CI: 27.94–86.52) and *S. longepedunculata* (41.2 mg/L; 95% CI: 29.90–82.83), whereas the LC₅₀ values were not estimable for *E. sphaerocephalus*, *F. speciosa*, *G. scabra*, *J. schimperiana*, *L. schimperi*, and *T. schimperiana* due to insufficient mortality (< 30–50% maximum mortality, according to standard probit criteria). The probit slopes ranged from 0.9 ± 0.7 to 2.5 ± 0.66, with low *X*^2^ goodness-of-fit values (≤ 1.02), indicating acceptable model fit (Table 3).

**Table 3:** Probit analysis of the effects of plant extracts against *Anopheles arabiensis*

Plant species	LC₅₀ (mg/L) (95% CI)	LC₉₀ (mg/L) (95% CI)	Slope ± SE	*χ*^2^ (*df* = 1)
*A. sisalana*	21.8 (14.8–29.1)	69.5 (45.9–215.7)	2.5 ± 0.66	1.02
*C. macrostachyus*	42.9 (27.2–405.8)	N/E	1.5 ± 0.6	0.09
*E. sphaerocephalus*	N/E	N/E	N/E	N/E
*F. speciose*	N/E	N/E	N/E	N/E
*F. thonningii*	39.5 (27.94–86.52)	N/E	2.0 ± 0.6	0.05
*G. scabra*	N/E	N/E	N/E	N/E
*J. schimperiana*	N/E	N/E	N/E	N/E
*L. schimperi*	N/E	N/E	N/E	N/E
*M. ferruginea*	19.1 (4.13–30.67)	N/E	1.5 ± 0.6	0.80
*M. foetida*	15.3 (0.94–24.39)	N/E	1.5 ± 0.7	0.20
*S. longepedunculata*	41.2 (29.90–82.83)	N/E	2.2 ± 0.7	0.50
*T. schimperiana*	N/E	N/E	N/E	N/E

LC₅₀ and LC₉₀ were estimated via probit regression. LC = lethal concentration; CI = confidence interval; N/E = not estimable due to < 30–50% maximum mortality. *χ*^2^ = Chi-square goodness-of-fit test (*df* = 1); *df* = degrees of freedom

### Adulticidal activity of 80% methanol plant extracts against *Aedes aegypti*

All the tested plant extracts exhibited varying adulticidal activities against *Ae. aegypti*, with mortality increasing in a concentration-dependent manner (Table 4). At 52.63 mg/L, *S. longepedunculata* achieved moderate mortality (82 ± 1.1%), followed by *M. ferruginea* (71 ± 0.8%) and *M. foetida* (69 ± 1.1%), while *G. scabra* (17 ± 0.1%) and *F. speciosa* (24 ± 0.7%) showed lower efficacy. Consistent increases in mortality across concentrations indicated a clear dose–response relationship; *A. sisalana* increased from 11 ± 0.8% at 13.16 mg/L to 26 ± 1.1% at 26.32 mg/L and 61 ± 1.2% at 52.63 mg/L, whereas *M. ferruginea* increased from 27 ± 0.8 to 71 ± 0.8%. One-way ANOVA confirmed highly significant differences among the concentrations for all the species (*p* < 0.001).

**Table 4:** Adulticidal activity of methanol plant extracts against *Aedes aegypti*

Plant species	Conc. (mg/L)	Mortality % (Mean ± SD)	ANOVA *F* (*df*), *p*-value
*A. sisalana*	13.16	11 ± 0.8ᵃ	*F* (2,6) = 418.6, *p* < 0.001
	26.32	26 ± 1.1ᵇ	
	52.63	61 ± 1.2ᶜ	
*C. macrostachyus*	13.16	10 ± 0.8ᵃ	*F* (2,6) = 216.5, *p* < 0.001
	26.32	29 ± 1.5ᵇ	
	52.63	38 ± 1.1ᶜ	
*E. sphaerocephalus*	13.16	14 ± 1.1^a^	*F* (2,6) = 66.6, *p* < 0.001
	26.32	21 ± 0.8^b^	
	52.63	29 ± 1.1^c^	
*F. speciosa*	13.16	10 ± 0.7a	*F* (2,6) = 64.7, *p* < 0.001
	26.32	20 ± 1.4^b^	
	52.63	24 ± 0.7^c^	
*F. thonningii*	13.16	6 ± 1.1ᵃ	*F* (2,6) = 287.2, *p* < 0.001
	26.32	9 ± 1.1^a^	
	52.63	40 ± 0.4^c^	
*G. scabra*	13.16	5 ± 1.1^a^	*F* (2,6) = 68.8, *p* < 0.001
	26.32	12 ± 0.7^b^	
	52.63	17 ± 0.1^c^	
*J. schimperiana*	13.16	17 ± 0.8^a^	*F* (2,6) = 55.8, *p* < 0.001
	26.32	22 ± 1.1^a^	
	52.63	32 ± 0.7^b^	
*L. schimperi*	13.16	8 ± 0.7^a^	*F* (2,6) = 216.7, *p* < 0.001
	26.32	20 ± 0.7^b^	
	52.63	31 ± 0.8^c^	
*M. ferruginea*	13.16	27 ± 0.8^a^	*F* (2,6) = 622.7, *p* < 0.001
	26.32	49 ± 1.4^b^	
	52.63	71 ± 0.8^c^	
*M. foetida*	13.16	23 ± 0.8^a^	*F* (2, 6) = 322.1, *p* < 0.001
	26.32	36 ± 0.7^b^	
	52.63	69 ± 1.1c	
*S. longepedunculata*	13.16	39 ± 1.5ᵃ	*F* (2,6) = 484.3, *p* < 0.001
	26.32	43 ± 1.4ᵃ	
	52.63	82 ± 1.1ᵇ	
*T. schimperiana*	13.16	9 ± 1.2^a^	*F* (2,6) = 173.1, *p* < 0.001
	26.32	18 ± 1.1^b^	
	52.63	35 ± 1.4^c^	
Negative control	5% DMSO	0	
Positive control	0.03% deltamethrin	97–98	N/A

Values are expressed as the means ± SDs of three replicates. One-way ANOVA followed by Tukey’s HSD test was used for comparisons. Different lowercase superscripts (a–c) within columns indicate significant differences in mortality concentration (*p* < 0.05); means sharing the same letter are not significantly different. mg/L = milligrams per liter; SD = standard deviation; *df* = degrees of freedom; N/A = not applicable

### Probit analysis and lethal concentrations against *Aedes aegypti*

Probit analysis revealed significant variability in adulticidal activity against *Ae. aegypti* (Table 5). *M. ferruginea* was most potent (LC₅₀ = 12.5 mg/L; 95% CI, 0.006–21.27), followed by *S. longepedunculata* (LC₅₀ = 22.4 mg/L; 95% CI, 12.47–33.07), *M. foetida* (LC₅₀ = 32.2 mg/L; 95% CI, 21.73–62.87), and *A. sisalana* (LC₅₀ = 42.7 mg/L; 95% CI, 32.1–75.5). The L₅₀ values were not estimable (N/E) for *C. macrostachyus*, *E. sphaerocephalus*, *F. speciosa*, *G. scabra*, *J. schimperiana*, and *L. schimperi* because these extracts produced insufficient mortality (maximum < 30–50%), preventing reliable estimation of the probit model.

**Table 5:** Probit analysis of the effects of plant extracts against *Aedes aegypti*

Plant species	LC₅₀ (mg/L) (95% CI)	LC₉₀ (mg/L) (95% CI)	Slope ± SE	*χ*^2^ (*df* = 1)
*A. sisalana*	42.7 (32.1–75.5)	135.2 (76.1–804.6)	2.56 ± 0.7	0.24
*C. macrostachyus*	N/E	N/E	N/E	N/E
*E. sphaerocephalus*	N/E	N/E	N/E	N/E
*F. speciose*	N/E	N/E	N/E	N/E
*F. thonningii*	N/E	N/E	N/E	N/E
*G. scabra*	N/E	N/E	N/E	N/E
*J. schimperiana*	N/E	N/E	N/E	N/E
*L. schimperi*	N/E	N/E	N/E	N/E
*M. ferruginea*	12.5 (0.006–21.27)	N/E	1.3 ± 0.6	0.30
*M. foetida*	32.2 (21.73–62.87)	N/E	1.8 ± 0.6	0.90
*S. longepedunculata*	22.4 (12.47–33.07)	104.2 (56.3–1390)	1.9 ± 0.6	2.40
*T. schimperiana*	N/E	N/E	N/E	N/E

LC₅₀ and LC₉₀ were estimated via probit regression. LC = lethal concentration; CI = confidence interval; N/E = not estimable due to < 30–50% maximum mortality. *χ*^2^ = Chi-square goodness-of-fit test (*df* = 1); *df* = degrees of freedom

### Repellent results

#### Essential oil yields

Hydrodistillation of 1000 g of fresh plant material yielded 17.0–21.0 mL of essential oil, corresponding to 1.7–2.1% (v/w) essential oil (Table 6). *S. longepedunculata* roots presented the highest oil yield (2.10%), followed by *M. foetida* leaves (1.95%).

**Table 6:** Essential oil yields from the hydrodistillation of selected Ethiopian medicinal plants

Plant species	Part used	Plant material (g)	Oil yield (ml)	Yield (% v/w)
*F thonningii*	leaf	1000	17.5	1.75
*G. scabra*	Leaf	1000	19	1.90
*M. foetida*	Leaf	1000	19.5	1.95
*S. longepedunculata*	Root	1000	21	2.10

Fresh weight basis; hydrodistillation (Clevenger apparatus, 3 h)

#### Repellent activity of essential oils of four selected plant species against *Anopheles arabiensis*

Table 7 summarizes the repellent activities of the essential oils from *F. thonningii*, *G. scabra*, *M. foetida*, and *S. longepedunculata* at concentrations of 2.5%, 5%, and 10% against adult mosquitoes. Repellent efficacy generally increased with increasing concentration for *M. foetida*, *F. thonningii*, and *S. longepedunculata*. Among the tested oils, *M. foetida* exhibited moderate repellency (80.0%) at 10%, followed by *F. thonningii* (73.0%) and *S. longepedunculata* (68.0%). Significant concentration-dependent effects were observed for these three oils (*p* < 0.05). Although *S. longepedunculata* showed a lower maximum repellency than *M. foetida*, it exhibited the lowest ED₅₀ value, indicating greater potency at lower concentrations. In contrast, *G. scabra* demonstrated weak repellent activity, with a maximum repellency of 26.7% at 10%, and concentration effects were not statistically significant (*p* > 0.05). The negative control (99% ethanol) showed no repellent activity, whereas the positive control (10% DEET) provided complete protection (100% repellency) throughout the observation period, confirming the validity of the bioassay.

**Table 7:** Repellent activity of selected plant extracts against *Anopheles arabiensis*

Plant species	Conc. (%)	Mean bites (± SD)	Repellency (%)	ED₅₀ (%) (95% CI)	ED₉₉ (%) (95% CI)	*F* value	*P*-value
*F. thonningii*	2.5	8.0 ± 1.0	46.6	4.9(3.9–6.1)	16(12.1–39.8)	(*F* (2, 6) = 7.234	0.025
	5	6.0 ± 1.0	60.0				
	10	4.0 ± 1.0	73.0				
*G. scabra*	2.5	13.7 ± 0.6	8.7	N/E	N/E	(*F* (2, 6) = 4.464	0.065
	5	12.0 ± 1.5	18.0				
	10	11.0 ± 1.0	26.7				
*M. foetida*	2.5	7.7 ± 1.1	48.0	4.2(3.3–5.4)	21.4(13.9–47.8)	(*F* (2, 6) = 10.918	0.01
	5	4.7 ± 1.5	69.0				
	10	3.0 ± 1.0	80.0				
*S. longepedunculata*	2.5	10.0 ± 1.0	33.0	3.9(3.1–4.9)	14.6(10.2–27.9)	(*F* (2, 6) = 29.524	0.001
	5	6.0 ± 1.0	60.0				
	10	4.7 ± 0.6	68.0				
Negative control	Ethanol- 99	N/A	0	N/A	N/A	N/A	N/A
Positive control	DEET- 10	N/A	100	N/A	N/A	N/A	N/A

Values are presented as the mean number of mosquito bites ± standard deviation (SD) and percentage repellency. Repellency (%) was calculated relative to the negative control. ED₅₀ and ED₉₉ represent the effective doses required to achieve 50% and 99% repellency, respectively, with corresponding 95% confidence intervals (CIs). *F* values were obtained via one-way analysis of variance (ANOVA) to compare mean bite counts among concentrations for each plant extract. A *p*-value < 0.05 was considered statistically significant. N/E = not estimable due to an insufficient repellency response within the tested concentration range. N/A = not applicable

### Complete protection time of essential oils from selected plants against *Anopheles arabiensis*

The effects of the selected plant extracts on CPT against adult *An. arabiensis* are presented in Table 8. *F. thonningii* showed statistically significant differences in CPT across concentrations (log-rank *χ*^2^ = 6.9, *p* = 0.03). The median CPT increased from 2 min (95% CI: 0.4–3.6) at 2.5% to 6 min (95% CI: 4.4–7.6) at 10%. *M. foetida* showed relatively low repellency among the tested species and also exhibited significant concentration-dependent differences in CPT (log-rank *χ*^2^ = 8.8, *p* = 0.012). The median CPT increased from 2 min (95% CI: 0.4–3.6) at 2.5% to 7 min (95% CI: 3.8–10.2) at 10%, indicating a dose–response relationship.

**Table 8:** Complete protection time of selected plant extracts against *Anopheles arabiensis*

Plant name	Conc. (%)	Median CPT (min) (95% CI)	Mean CPT ± SE (min)	*X*^2^ (*df* = 2)	*P*-value
*F, thonningii*	2.5	2 (0.4–3.6)	2.0 ± 0.5	6.9	0.030
	5	4 (0.8–7.0)	4.0 ± 1.1		
	10	6 (4.4–7.6)	6.0 ± 0.5		
*G. scabra*	2.5	0 (0–2.0)	0.5 ± 0.3	2.1	0.350
	5	1 (0–2.6)	1.0 ± 0.5		
	10	2 (0.4–3.6)	2.0 ± 0.5		
*M. foetida*	2.5	2 (0.4–3.6)	2.0 ± 0.5	8.8	0.012
	5	5 (1.8–8.2)	4.6 ± 0.8		
	10	7 (3.8–10.2)	6.6 ± 0.8		
*S. longepedunculata*	2.5	4 (0–6.0)	3.3 ± 0.6	5.1	0.076
	5	4 (2.4–5.6)	4.3 ± 0.8		
	10	7 (3.8–10.2)	6.6 ± 0.8		

Values are presented as median CPTs with 95% confidence intervals (CIs) and means ± standard errors (SEs). CPT (complete protection time) represents the duration from the application of the plant extract until the first mosquito bite was observed. Differences among the concentrations of each plant extract were analyzed via the Kaplan–Meier survival method followed by the log-rank test (*df* = 2). A *p*-value < 0.05 was considered statistically significant

For *S. longepedunculata*, CPT tended to increase with concentration, but the effect was not statistically significant (log-rank *χ*^2^ = 5.1, *p* = 0.076). The median CPT ranged from 4 min at 2.5% to 7 min at 10%. For *G. scabra*, CPT values remained low across all concentrations, and no statistically significant differences were detected (log-rank *χ*^2^ = 2.1, *p* = 0.350), indicating minimal repellent activity under the tested conditions.

### Repellent activity of essential oils of four selected plant species against *Aedes aegypti*

Table 9 presents the repellency data for essential oils from *F. thonningii*, *G. scabra*, *M. foetida*, and *S. longepedunculata* at concentrations of 2.5%, 5%, and 10%, including the mean number of mosquito bites (mean ± SD), percentage repellency, effective doses (ED₅₀ and ED₉₉ with 95% confidence intervals), and one-way ANOVA results (*F*- and *p* values) for concentration effects. *S. longepedunculata* showed measurable repellency, with 2.3 ± 0.6 mean bites (86.7% protection) at 10% (*F* (2,6) = 45.404, *p* = 0.001; ED₅₀ = 3.5%, 95% CI: 2.8–4.4). *F. thonningii* reduced bites to 3.6 ± 0.6 at 10% (79.0% protection; *F* (2,6) = 20.191, *p* = 0.002; ED₅₀ = 4.6%, 95% CI: 3.8–5.8). *M. foetida* achieved 63.5% protection at 10% with moderate effects (*F* (2,6) = 9.644, *p* = 0.013; ED₅₀ = 5.0%, 95% CI: 3.9–6.7). *G. scabra* was the least effective (34.6% maximum repellency at 10%; *F* (2,6) = 3.733, *p* = 0.088; ED₅₀ = 9.12%, 95% CI: 6.8–14.9).

**Table 9:** Repellent activity of selected plant extracts against *Aedes aegypti*

Plant species	Conc. (%)	Mean bite (± SD)	Repellency (%)	ED₅₀ (%) (95% CI)	ED₉₉ (%) (95% CI)	*F* value	*P* value
*F. thonningii*	2.5	9.6 ± 1.2	44.0	4.6(3.8–5.8)	17.9(11.8–36.4)	(*F* (2, 6) = 20.191	0.002
	5	7.6 ± 1.5	56.0				
	10	3.6 ± 0.6	79.0				
*G. scabra*	2.5	14.3 ± 1.1	17.0	9.12(6.8–14.9)	N/E	(*F* (2, 6) = 3.733	0.088
	5	13.6 ± 1.5	21.0				
	10	11.3 ± 1.5	34.6				
*M. foetida*	2.5	11.0 ± 1.0	36.0	5.0(3.9–6.7)	22.8(14.8–52.3)	(*F* (2, 6) = 9.644	0.013
	5	8.3 ± 1.1	52.0				
	10	6.3 ± 1.5	63.5				
*S. longepedunculata*	2.5	11.0 ± 1.0	36.0	3.5(2.8–4.4)	13.2(9.4–24.8)	(*F* (2, 6) = 45.404	0.001
	5	6.3 ± 1.5	63.5				
	10	2.3 ± 0.6	86.7				
Negative control	Ethanol- 99	N/A	0	N/A	N/A	N/A	N/A
Positive control	DEET- 10	N/A	100	N/A	N/A	N/A	N/A

Values are presented as the mean number of mosquito bites ± standard deviation (SD) and percentage repellency. Repellency (%) was calculated relative to the negative control. ED₅₀ and ED₉₉ represent the effective doses required to achieve 50% and 99% repellency, respectively, with corresponding 95% confidence intervals (CIs). *F* values were obtained via one-way analysis of variance (ANOVA) to compare mean bite counts among concentrations for each plant extract. A *p*-value < 0.05 was considered statistically significant. N/E = not estimable due to insufficient repellency response within the tested concentration range. N/A = not applicable

### Complete protection time of essential oils from selected plants against *Aedes aegypti*

Reporting Table 10, *F. thonningii* showed a few increases in median CPT from 1 min (95% CI: 0–2.6) at 2.5% to 3 min (95% CI: 1.4–4.6) at 10%. However, differences among concentrations were not statistically significant (log-rank *χ*^2^ = 3.91, *p* = 0.140); similarly, *G. scabra* showed minimal protective efficacy across all concentrations. Median CPT was 0 min at both 2.5% and 5%, and only 1 min (95% CI: 0–1.0) at 10%. The log-rank test revealed no significant differences among treatments (*χ*^2^ = 4.57, *p* = 0.100), confirming weak and inconsistent repellency.

**Table 10:** Complete protection time of selected plant extracts against *Aedes aegypti*

Plant name	Conc. (%)	Median CPT (min) (95% CI)	Mean CPT ± SE (min)	*X*^*2*^ (*df* = 2)	*p*-value
*F, thonningii*	2.5	1 (0–2.6)	1.0 ± 0.6	3.91	0.140
	5	2 (0–7.8)	2.0 ± 1.2		
	10	3 (1.4–4.6)	3.0 ± 0.8		
*G. scabra*	2.5	0 (0–0)	0.0 ± 0.0	4.57	0.100
	5	0 (0–0)	0.0 ± 0.0		
	10	0 (0–1.0)	1.0 ± 0.5		
*M. foetida*	2.5	0 (0–1.5)	1.5 ± 0.3	10.2	0.006
	5	1 (0–3.0)	3.6 ± 0.8		
	10	4 (0.8–7.2)	2.0 ± 0.5		
*S. longepedunculata*	2.5	2 (0.4–3.6)	2.0 ± 0.5	8.1	0.017
	5	4 (0.8–7.2)	3.6 ± 0.8		
	10	7 (3.8–10.2)	6.7 ± 0.8		

Values are presented as median CPTs with 95% confidence intervals (CIs) and means ± standard errors (SEs). CPT (complete protection time) represents the duration from the application of the plant extract until the first mosquito bite was observed. Differences among the concentrations of each plant extract were analyzed via the Kaplan–Meier survival method followed by the log-rank test (*df* = 2). A *p* value < 0.05 was considered statistically significant

In contrast, *M. foetida* showed a statistically significant concentration-dependent effect on CPT (*χ*^2^ = 10.2, *p* = 0.006). Median CPT increased from 0 min (95% CI: 0–1.5) at 2.5% to 4 min (95% CI: 0.8–7.2) at 10%. Similarly, *S. longepedunculata* showed an increase in CPT with increasing concentration (*χ*^2^ = 8.1,* p* = 0.017), with median CPT increasing from 2 min (95% CI: 0.4–3.6) at 2.5% to 7 min (95% CI: 3.8–10.2) at 10%.

### Preliminary phytochemical screening of biologically active plant species

Qualitative phytochemical analysis (Table 11) revealed the presence of diverse secondary metabolites in the methanolic extracts of the tested plants. Alkaloids, flavonoids, terpenoids, tannins/phenolic compounds, and saponins were detected most consistently. *M. ferruginea* presented the broadest profile, with positive results for alkaloids, flavonoids, tannins/phenolic compounds, and terpenoids. *A. sisalana* contains alkaloids, tannins/phenolics, and terpenoids, whereas *M. foetida* contains alkaloids, saponins, terpenoids, and tannins/phenolics. *S. longepedunculata* is particularly rich in flavonoids, saponins, tannins/phenolic compounds, terpenoids, and alkaloids.

**Table 11:** Results of preliminary phytochemical screening of selected plant species used in this study

Plant species	Saponins	Tannins	Flavonoids	Alkaloids	Terpenoids	Phenols
*A. sisalana*	−	+	+	+	+	+
*M. ferruginea*	−	+	+	+	+	+
*M. foetida*	+	+	+	+	+	+
*S. longepedunculata*	+	+	+	+	+	+

(+) implies positive and (−) implies negative

### Thin-layer chromatography profiling of extracts using *n*-butanol:glacial acetic acid:water (4:1:1, v/v/v)

**Fig. 1:**
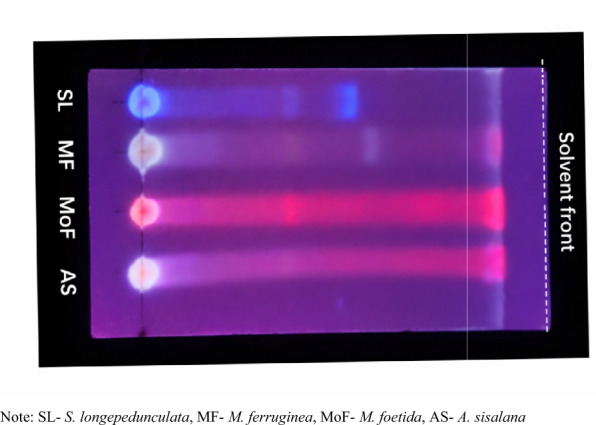
TLC chromatograms of 80% methanol crude extracts of *S. longepedunculata*, *M. ferruginea*, *M. foetida*, and *A. sisalana* developed using *n*-butanol:glacial acetic acid:water (4:1:1, v/v/v) and visualized under UV light (254 nm). SL—*S. longepedunculata*, MF—*M. ferruginea*, MoF—*M. foetida*, AS—*A. sisalana*

Silica gel TLC profiling of *S. longepedunculata*, *M. ferruginea*, *M. foetida*, and *A. sisalana* extracts was conducted using *n*-butanol:glacial acetic acid:water (4:1:1, v/v/v) as the mobile phase in the solvent system (Fig. 1). Visualization under UV light (254 nm) revealed distinct fluorescent bands along the plate from the origin (bottom) to the solvent front (top). *S. longepedunculata* and *M. ferruginea* presented prominent bands in the upper regions of the chromatogram, whereas *M. foetida* and *A. sisalana* presented well-resolved bands at intermediate *R*_f_ values.

### Retention factor values from thin-layer chromatography analysis

TLC analysis of the plant extracts obtained using *n*-butanol:glacial acetic acid:water (4:1:1, v/v/v) (Table 12) revealed distinct, well-resolved spot patterns, indicating variations in phytochemical composition among the species. *S. longepedunculata* presented two bands with *R*_f_ values of 0.10 and 0.41, whereas *M. ferruginea* presented two major bands at *R*_f_ values of 0.14 and 0.45. *M. foetida* displayed a single prominent major band at *R*_f_ 0.75, whereas *A. sisalana* produced two major bands at *R*_f_ 0.29 and 0.83.

**Table 12:** *R*_f_ values of 80% methanol extracts obtained with *n*-butanol:glacial acetic acid:water (4:1:1, v/v/v) and visualized under UV light (254 nm)

Plant extract	Number of bands	*R*_f_ value (mm)
*S. longepedunculata*	2	0.10, 0.41
*M. ferruginea*	2	0.14, 0.45
*M. foetida*	1	0.75
*A. sisalana*	2	0.29, 0.83

The number of bands indicates the spot number; *R*_f_ = distance traveled by the compound/distance traveled by the solvent front; mm = millimeter

### Thin-layer chromatography profiling of extracts using *n*-butanol:chloroform (7:3, v/v)

**Fig. 2:**
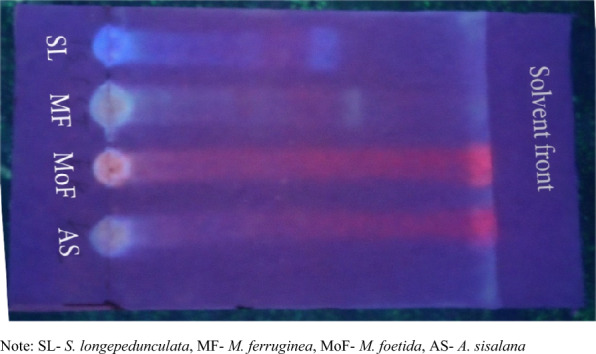
TLC chromatograms of 80% methanol crude extracts of *S. longepedunculata*, *M. ferruginea*, *M. foetida*, and *A. sisalana* developed using *n*-butanol:chloroform (7:3, v/v) and visualized under UV light (366 nm). SL—*S. longepedunculata*, MF—*M. ferruginea*, MoF—*M. foetida*, AS—*A. sisalana*

TLC analysis of the plant extracts obtained using the second solvent system (*n*-butanol:chloroform, 7:3, v/v) revealed distinct banding patterns compared with those of the first system (Fig. 2). Notable differences included higher *R*_f_ values, increased fluorescence intensity, and improved resolution of less polar compounds. These features were particularly evident in the upper regions of the plates for *M. foetida* and *A. sisalana* under UV light at 366 nm.

### Retention factor values from thin-layer chromatography analysis

TLC analysis of the plant extracts using *n*-butanol: chloroform (7:3, v/v) (Table 13) revealed distinct separation patterns, indicating variations in phytochemical composition among the species. *S. longepedunculata* presented two major bands with *R*_f_ values of 0.12 and 0.39, whereas *M. ferruginea* presented two major bands with *R*_f_ values of 0.16 and 0.43. *M. foetida* presented a single prominent band at *R*_f_ = 0.78, whereas *A. sisalana* presented two bands at *R*_f =_ 0.31 and 0.85, suggesting the presence of both moderately polar and less polar constituents.

**Table 13:** *R*_f_ values of 80% methanol extracts obtained via *n*-butanol:chloroform (7:3, v/v)

Plant extract	Number of bands	*R*_f_ value (mm)
*S. longepedunculata*	2	0.12, 0.39
*M. ferruginea*	2	0.16, 0.43
*M. foetida*	1	0.78
*A. sisalana*	2	0.31, 0.85

## Discussion

This study demonstrated that methanol extracts and essential oils from selected Ethiopian medicinal plants exhibit measurable but variable adulticidal and repellent activities against *An. arabiensis* and *Ae. aegypti* under laboratory conditions. Mortality responses and protection times differed across plant species and concentrations, indicating dose-dependent effects, although the magnitude of activity was generally moderate.

Among the tested species, *A. sisalana*, *M. foetida*, *S. longepedunculata*, and *M. ferruginea* presented relatively high adulticidal activity against *An. arabiensis*. For example, *A. sisalana* achieved up to 80% mortality at the highest tested concentration (52.63 mg/L), with an estimated LC₅₀ of 21.8 mg/L. However, these results should be interpreted with caution, as the range of tested concentrations was limited and some confidence intervals were relatively wide, indicating uncertainty in the probit estimates. Previous studies reporting the larvicidal activity of *A. sisalana* against mosquito species such as *Culex quinquefasciatus* (*C. quinquefasciatus*), *Ae. aegypti* and *Anopheles stephensi* (*An. Stephensi*) support its insecticidal potential across life stages [24], and the present findings extend these observations to adult mosquitoes.

*Momordica foetida* exhibited notable adulticidal activity against *An. arabiensis,* with an LC₅₀ value of 15.3 mg/L. TLC profiling revealed a prominent band at Rf = 0.78, suggesting the presence of nonpolar or less polar constituents. However, due to the absence of reference standards, no specific compounds could be identified from the chromatographic analysis. Previous studies have reported the insecticidal potential of *M. foetida* and have proposed the involvement of secondary metabolites such as cucurbitacins and triterpenoids [25]. Nevertheless, these compounds were not directly detected in the present study, and any linkage between the observed bioactivity and particular phytochemical classes remains speculative. According to a previous study, these metabolites are associated with mechanisms such as disruption of feeding behavior and impairment of cellular integrity in insects [26].

*Millettia ferruginea* seed extracts show consistent adulticidal activity against *An. arabiensis*. Our immersion bioassay yielded an LC₅₀ = 19.1 mg/L. Moreover, another study reported topical LC₅₀ values of 46.03 mg/cm^2^ (1 h), 34.86 mg/cm^2^ (2 h), and 29.77 mg/cm^2^ (3 h) for laboratory strains, confirming their potency [27] despite the methodological and concentration differences reported in the present study. Previous studies have suggested that the activity of *M. ferruginea* is associated with isoflavonoids and related secondary metabolites, which disrupt enzymatic and membrane functions [28]. Earlier reports of its larvicidal and pupicidal effects on *An. arabiensis* [27]. These findings further confirmed the broad-spectrum efficacy of this approach across mosquito life stages.

Similarly, *S. longepedunculata* exhibited a clear dose–response relationship and relatively low LC values, indicating consistent toxicity under laboratory conditions. However, in some cases, the LC₉₉₀ values were not estimable, likely due to insufficient mortality at higher concentration thresholds, suggesting reduced efficacy at extreme doses or variability in response. TLC analysis revealed distinct bands at Rf values of 0.12 and 0.39, indicating the presence of multiple moderately polar phytochemical constituents. Preliminary phytochemical screening further confirmed the presence of major classes of secondary metabolites, supporting the chemical complexity of the extract. Nevertheless, both TLC and screening approaches are qualitative and do not permit definitive compound identification or direct attribution of biological activity.

Previous studies have reported that *S. longepedunculata* contains bioactive compounds such as phenolics, coumarins, and alkaloids [29], which may contribute to its insecticidal properties. These metabolites are associated with various mechanisms, including neurotoxicity and the disruption of respiratory processes in insects [30]. The coexistence of multiple constituents may also result in additive or synergistic interactions, thereby enhancing the overall insecticidal efficacy [31]. However, as these compounds were not directly identified in the present study, any linkage between specific phytochemicals and the observed adulticidal activity remains tentative. Therefore, further investigations using bioassay-guided fractionation and compound isolation are needed to identify the active compounds and clarify their modes of action.

In contrast, species such as *C. macrostachyus*, *F. speciosa*, and *G. scabra* exhibited limited adulticidal activity, with low mortality rates and, in several cases, an inability to estimate the LC₅₀ or LC₉₀ values. This finding is consistent with the observed low-dose response slopes and highlights the bioactivity variability among plant species.

Against *Ae. aegypti*, *S. longepedunculata,* and *M. ferruginea* produced measurable mortality. *S. longepedunculata* caused the highest mortality at the highest concentration tested, while *M. ferruginea* had lower LC_50_ values, indicating greater potency at lower exposure levels. Interpretation remains limited, however, because only a small number of concentrations were tested, and some treatments produced relatively low mortality.

In terms of repellency, the essential oils of *S. longepedunculata*, *M. foetida*, and *F. thonningii* produced moderate reductions in mosquito biting rates, reaching 86%, 80%, and 79%, respectively, during short exposure periods. However, the duration of complete protection was limited, ranging from 6 to 7 min. Although these oils exhibit measurable immediate behavioral effects, their protective efficacy is short-lived and may have limited practical application in their current, unformulated form. Early research reports that the repellency of *S. longepedunculata* aligns with reports on methyl salicylate and coumarins, which disrupt mosquito host seeking via olfactory interference [32]. Similar studies reported up to 100% repellency against *Anopheles gambiae s.l.* and *Culex quinquefasciatus* at 0.3–0.5 mg/mL, often surpassing DEET [33]. Similarly, *M. foetida* may contain volatile terpenoids that contribute to spatial repellency, whereas the activity of *F. thonningii* may be associated with secondary metabolites such as methyl ketones and alkaloids [34, 35]. Overall, the repellent efficacy of the essential oils appears to be influenced primarily by their volatility and interactions with mosquito olfactory receptors rather than by direct toxic effects, which may explain the relatively short duration of protection observed.

Several limitations should be considered when interpreting this study. First, adulticidal efficacy was evaluated using solution-based concentrations (mg/L) rather than surface doses (mg/cm^2^). Although approximate mg/cm^2^ conversions were provided in the Materials and methods section for reference, all efficacy parameters (LC₅₀, LC₉₀, and mortality) were reported using mg/L. Although this approach facilitated the preparation and comparison of test concentrations under laboratory conditions, the surface dose is generally considered a more relevant measure of exposure in contact bioassays. Consequently, the use of mg/L may limit direct comparisons with studies reporting results in mg/cm^2^ and may reduce the applicability of the findings to field conditions. Second, the limited number of test concentrations and the relatively low mortality responses observed for some extracts reduced the precision of the probit-derived LC₅₀ and LC₉₀ estimates, as reflected by wide confidence intervals and non-estimable values in some cases. Consequently, these toxicity estimates should be interpreted with caution. Third, all experiments were conducted under controlled laboratory conditions using crude plant extracts and essential oils, which may not fully reflect their effectiveness under field conditions, where environmental factors, mosquito behavior, and formulation stability can influence efficacy. In addition, the chemical composition of the essential oils was not characterized using advanced analytical techniques, such as gas chromatography–mass spectrometry (GC–MS), due to resource constraints. As a result, the specific bioactive compounds responsible for the observed repellent activity could not be identified. Future studies should therefore focus on comprehensive chemical characterization of these essential oils to identify and quantify their major constituents and assess their individual and synergistic contributions to mosquito repellency and insecticidal activity. Despite these limitations, the present study provides valuable preliminary evidence of the insecticidal and repellent potential of the tested plant species and underscores the need for further research involving expanded dose‒response assessments, formulation optimization, and field-based validation.

## Conclusion and recommendation

These findings indicate that crude extracts and essential oils from these indigenous Ethiopian plants could contribute to integrated vector management programs. This study provides preliminary evidence of measurable adulticidal and repellent activities of selected Ethiopian medicinal plants against *An. arabiensis* and *Ae. aegypti*, with the observed effects being generally moderate and variable across species. The crude extracts of *A. sisalana*, *M. foetida*, *M. ferruginea*, and *S. longepedunculata* presented relatively high adulticidal activity, whereas the essential oils of *S. longepedunculata* and *M. foetida* presented short-term repellency.

However, important limitations, including reliance on surrogate exposure measures, a limited concentration range, and laboratory-based conditions, limit the generalization of these findings. Future studies should prioritize bioassay-guided fractionation, identification of active compounds, improved formulations to increase residual activity, evaluation of safety for non-target organisms, and validation under field conditions. In summary, these preliminary findings suggest that indigenous Ethiopian plants hold promise as potential complementary components in integrated vector management strategies, although further optimization is needed before their practical implementation.

## Supplementary Information

Supplementary Material 1.

## Data Availability

No datasets were generated or analyzed during the current study.
