# Supplementary material for: Adulticidal and repellent activities of traditionally used Ethiopian plants against Anopheles arabiensis and Aedes aegypti
Source: Trop Med Health. 2026 Jul 15;54:130. doi: 10.1186/s41182-026-01029-y (PMC13374292; doi:10.1186/s41182-026-01029-y)

Uncropped Original TLC Plate Image

Full uncropped original Thin-Layer Chromatography (TLC) plate image for Figure 1. No gels or blots were used

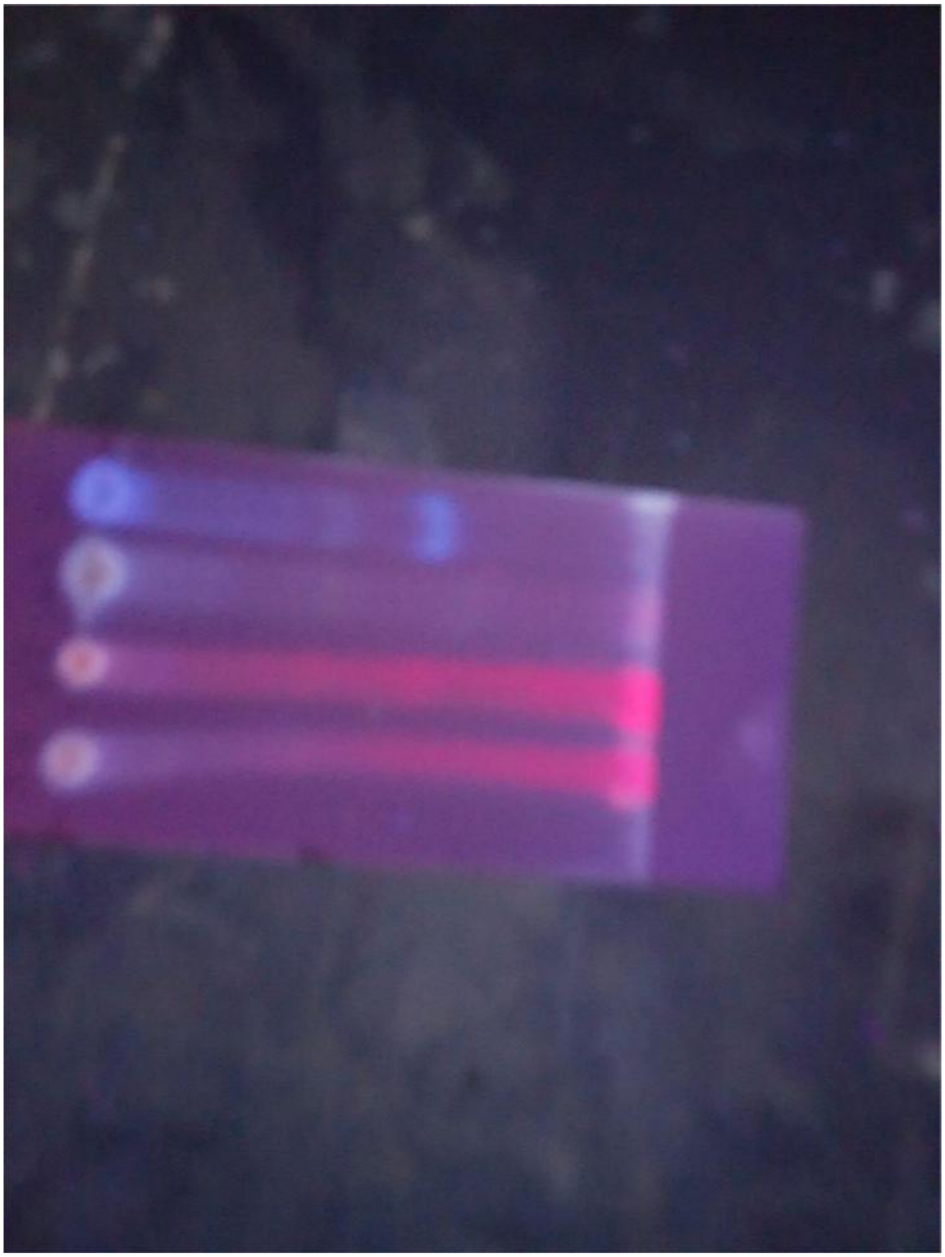

Full uncropped original Thin-Layer Chromatography (TLC) plate image for Figure 2. No gels or blots were used

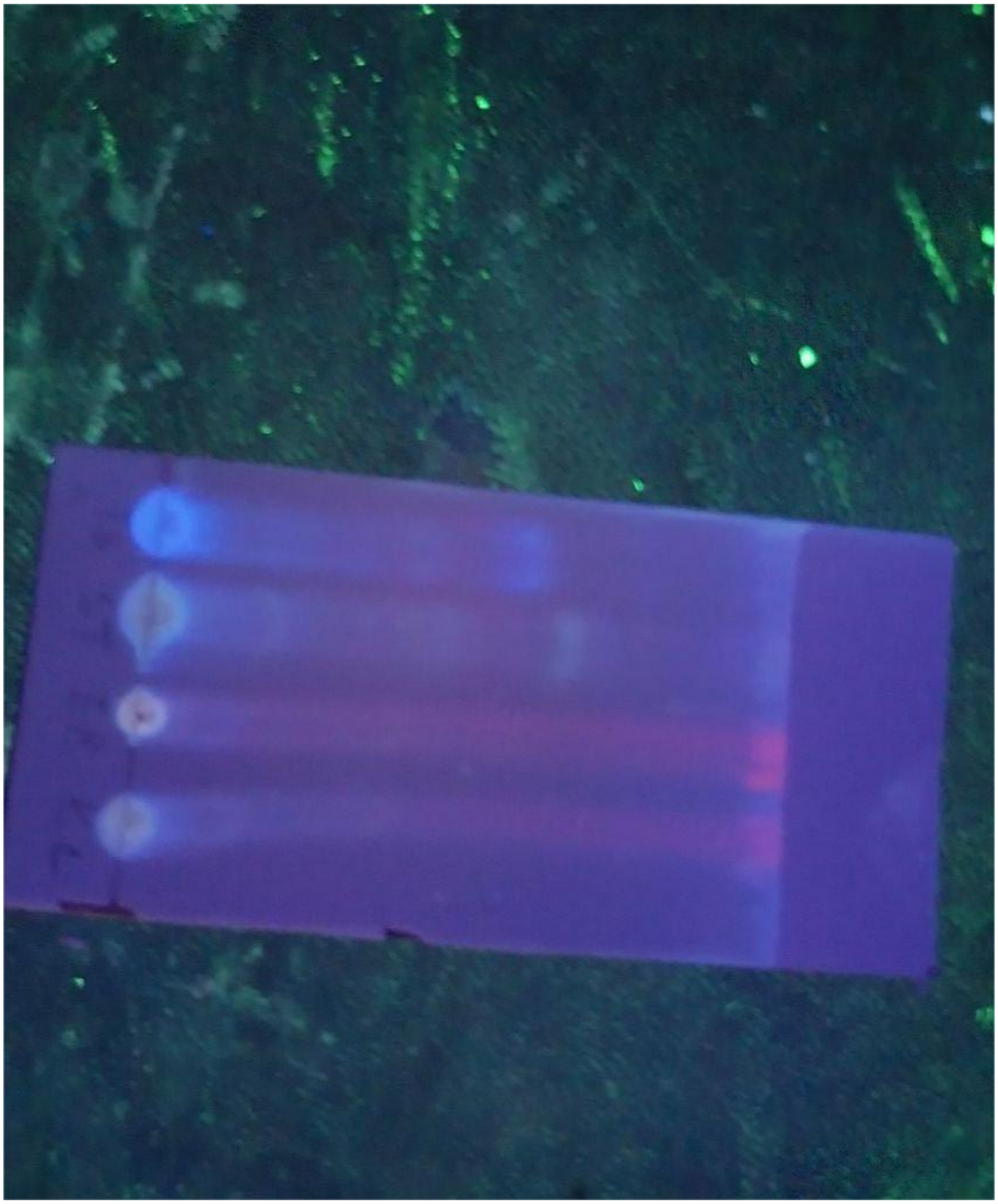

Supplement: Supplementary file 1 — Supplementary Material 1. [file 41182_2026_1029_MOESM1_ESM.pdf]
